# Can the presence of specialized addiction staff in primary health care increase the number of alcohol-related medical consultations – A controlled intervention study

**DOI:** 10.1016/j.abrep.2024.100526

**Published:** 2024-01-04

**Authors:** Tove Abrahamsson, Ester Magnusdottir, Jonas Berge, Åsa Lundvall, Agneta Öjehagen, Anders Håkansson

**Affiliations:** a Lund University, Faculty of Medicine, Department of Clinical Sciences, Psychiatry, Lund, Sweden; b Region Skåne, Center of Primary Care, Malmö, Sweden; c Skaraborg Hospital, Region Västra Götaland, Skövde, Sweden; d Region Skåne, Malmö Addiction Center/Competence Center Addiction, Malmö, Sweden

**Keywords:** Alcohol use disorder, Primary care, Early intervention

## Abstract

**Background:**

Few individuals with alcohol use disorders receive treatment. Primary care has been suggested as an arena for early treatment for these disorders.

**Aim:**

To evaluate whether the presence of a specialized addiction nurse can increase alcohol-related physician consultations in a primary care setting.

**Method:**

This controlled intervention study included one intervention and one control primary care unit in Malmö, Sweden. At the intervention unit, an addiction nurse experienced in alcohol use disorder treatment was present 20 h weekly for 12 months. At both units, an educational lecture on alcohol use disorders was given at study start. The outcome was physicians’ monthly number of alcohol-related diagnostic codes. Data were compared between intervention and control units using Poisson Regression. Eight statistical models were analyzed and Akaike information criterion was used to select the final model.

**Results:**

The intervention was significantly associated with an increased number of registered alcohol-related diagnostic codes (risk ratio 1.33, 95 confidence interval 1.08–1.62). However, in sensitivity analyses, such a slope effect was more uncertain and no step effect was seen. A significant association was seen between the educational lecture and an increase in the number of registered alcohol-related codes at the sites (risk ratio 2.47, 1.37–4.46).

**Conclusion:**

The presence of specialized addiction staff in a primary healthcare setting might increase the number of alcohol-related physician consultations in primary care, although more research is needed. An educational lecture about alcohol use disorders could be a simple but effective intervention to increase alcohol-related physician consultations in primary care.

## Highlights

• Primary care is under-utilized for the management of alcohol use disorders.
• Presence of an alcohol-oriented nurse may be promising in alcohol use management.
• More data is needed regarding effects of alcohol-specialist staff in primary care.
• A specialist lecture can increase primary care efforts in alcohol use disorders.

## 1 Introduction

Harmful use of alcohol, defined as “drinking that causes detrimental health and social consequences for the drinker, the people around the drinker and society at large, as well as patterns of drinking that are associated with increased risk of adverse health consequences”, has been estimated as the seventh leading risk factor for disease, disability and death in the world, and as the number one risk factor in the population aged 15–49 years. Around 3 million deaths (5,3% of all deaths) were attributable to harmful alcohol use in 2016 (World Health Organization (2018), 2018). Globally, 8.6 % of adult men and 1.7 % of adult women are estimated to suffer from alcohol use disorders (AUDs), with the highest prevalence rates seen in Northern Europe (including Russia) and North America (Carvalho, Heilig, Perez, Probst, & Rehm, 2019).

Despite the high prevalence rates, AUDs are often underdiagnosed and undertreated (Carvalho et al., 2019). A Swedish Government Official Report concluded that only about 20 % of individuals with alcohol dependence are reached by the health care system and that individuals with a harmful use of alcohol, but who do not meet the criteria for alcohol dependence, are reached to an even lesser degree (Socialdepartementet, 2011). Studies conducted in other countries have reported similar findings, with a minority of people with AUDs seeking and receiving treatment (Alonso et al., 2004, Carvalho et al., 2019, Cunningham and Breslin, 2004).

Patient-related factors, such as a lack of problem awareness or insufficient knowledge about treatment options, as well as the stigmatization of AUDs, are a part of the explanation for the low diagnostic and treatment prevalence of these disorders (Probst, Manthey, Martinez, & Rehm, 2015), but another important reason is that the primary health care (PHC) system in many countries is not adequately efficient in reaching and diagnosing these individuals (Carvalho et al., 2019). PHC is often the first point of contact for individuals seeking treatment for non-emergency health problems, and in a general population survey from the setting studied here, it has been demonstrated that a substantial proportion of respondents would prefer to recommend primary care as the point of contact for a potential significant other suffering from AUDs (Andréasson, Danielsson, & Wallhed-Finn, 2013). Furthermore, AUDs are often comorbid with or contributing causes of many common somatic and psychiatric disorders, and are thus more prevalent in PHC patients than in the general public (Rehm et al., 2016, Pilowsky and Wu, 2012, World Health Organization., 2018). PHC is therefore an important arena for early identification and initial treatment of people with AUDs (Carvalho et al., 2019, Rehm et al., 2016). Recommendations for the primary care management of AUDs typically include the importance to screen for alcohol problems in broader groups of patients, and to apply brief interventions (Zhang, Li, & Lee, 2017). It also has been increasingly emphasized that treatment of AUDs in primary care settings should include integrated strategies, which do not only include pharmacological treatment, but also different psycho-therapeutic programs, typically brief interventions and other motivational interventions (Hyland, McDowell, Bain, Huskamp, & Busch, 2023).

While several guidelines recommend general screening for AUDs in PHC, studies show that the rates of screening are often low (Rehm et al., 2016). A Swedish study found that general practitioners (GPs) perceived their own ability to counsel patients about alcohol habits to be poorer than counselling for other lifestyle behaviors (Geirsson, Bendtsen, & Spak, 2005). Another Swedish study found that while a large majority of both GPs and nurses found it very important to ask patients about alcohol use, only 50 % of the GPs and 28 % of the nurses frequently addressed alcohol habits with their patients (Holmqvist et al., 2008).

To investigate a method for increased screening for and treatment of AUDs, the main aim of this study was to evaluate whether the presence of a specialized addiction resource, i.e. a nurse with competence and experience in treating individuals with alcohol problems, would increase the alcohol-related physician consultations (i.e. consultations regarding AUDs) at a primary health care center (PHCC). We hypothesized that the presence of the addiction nurse would increase the tendency amongst the physicians and other ordinary staff at the IPHCC to screen and initiate treatment for AUDs, thus leading to an increase in alcohol-related physician consultations at the intervention primary health care center (IPHCC) as compared to a control primary health care center (CPHCC).

## 2 Methods

### 2.1 Study design

This was a controlled intervention study. Two PHCCs in Malmö, Sweden were included; one as the IPHCC and one as a CPHCC. The IPHCC was provided with a nurse from the Malmö Addiction Center, with competence and experience in treating people with AUDs. The nurse worked at the IPHCC 20 h per week during the 12 months that the study was conducted. The intervention also included a psychiatrist specialized in substance use disorders, as a consultant for the study nurse. Physicians working at the IPHCC (as well as all the other staff members) thus were informed about the fact that if they encountered patients who were diagnosed with AUDs, these patients could therefore easily be referred to the study nurse, such that they now had access to specialized on-site addiction consultations. The nurse provided the following treatments and interventions: 1) brief counselling. 2) Motivational Enhancement Therapy (MET) (Diclemente, Corno, Graydon, Wiprovnick, & Knoblach, 2017). 3) blood tests for alcohol markers (primarily phosphatidylethanol (PEth) and gamma-glutamyltransferase (GT). 4) if needed screening for comorbid psychiatric disorders using the Mini International Neuropsychiatric Interview (MINI).

The study was introduced to the staff at both the IPHCC and the CPHCC through an educational lecture, including diagnostics and treatment. More specifically, the lecture addressed the importance of screening and the particular importance of primary care units for the detection of hazardous alcohol consumption, specific screening tools, diagnostic criteria, biomarkers, psychological treatment methods, and indications for and specific considerations to address in pharmaceutical treatment. In addition, a brief information about the present study was provided. The reason for providing this educational intervention at both units, was to allow for an isolation of the effect of the actual study intervention at the IPHCC compared to the CPHCC where that intervention did not take place. The lecture was provided by the study nurse, together with a psychiatrist specialized in substance use disorders, and given twice, so that the entire staff received the information. The lecture was provided during lunch time at each primary care unit, in parallel with a lunch offered to the staff in order to facilitate their presence. The lecture – a prepared presentation and possibilities to ask questions – took around 45 min.

The study was approved by the Regional Ethical Review Board in Lund, Sweden. Since this was a register-based study without personal data, no informed consent was collected from the study individuals.

### 2.2 Setting

Both PHCCs were located in the inner-city area of Malmö, Sweden. The study started on the 13th of May 2015 at the IPHCC and on the 26th of May 2015 at the CPHCC, and the study period then continued for 12 months at each PHCC.

At the IPHCC, there were a total of 32 staff members, of whom eight were physicians, and at the CPHCC, there were 37 staff members, of whom 12 were physicians. For the purpose of the study, and with the assistance of the research organization within the primary care organization in the region, a control unit was chosen. The choice of the CPHCC was done in order to avoid apparent differences in the socio-demographic distribution of the units’ uptake areas, and based on an overall description of both units as having both socially more advantaged and socially more challenging areas within their uptake areas. Also, the research group had previous experience of project work at the CPHCC, which also facilitated the choice of this unit. The IPHCC had 9,588 listed patients and the CPHCC had 15,054. For details on age and gender distribution of the patients, see Table 1. At the start of the intervention, the Care Need Index (CNI) was 1.31 for the IPHCC and 1.26 for the CPHCC. The CNI describes the risk of developing illness based on socioeconomic factors. A higher CNI implies a higher risk of morbidity in the population (and thus a potentially higher workload for the PHCC). The ACG was 0.94 for the IPHCC and 0.84 for the CPHCC. The ACG is a diagnosis-based measure of morbidity that can be used to describe a potential need of healthcare resources in a population. A higher ACG implies a higher degree of total morbidity in the population.

**Table 1:** Number of listed patients shown with age and gender distribution at the two PHCCs.

	Intervention PHCC	Control PHCC	*p* value
Ages 0–18	2,095, 22 % (46 % female)	2,827, 19 % (49 % female)	<0.04
Ages 19–74	6,858, 72 % (43 % female)	11,674, 78 % (50 % female)	<0.01
Ages > 75	635, 7 % (55 % female)	553, 4 % (63 % female)	<0.01
Total	9,588, 100 %	15,054, 100 %	

### 2.3 Data sources

The data was extracted from the electronic health records database by an administrator at each PHCC. We collected data on all alcohol-related ICD-10 diagnosis codes registered as a consultation diagnosis by physicians at each PHCC for each monthly period during the 12 months before and after the study start. It was not possible to extract data on the number of alcohol-related ICD-10 diagnosis codes registered by other staff at the IPHCC, i.e. including those registered by the study nurse.

We furthermore collected data from the electronic health records database on the total number of patient consultations (i.e. not only alcohol-related consultations), including physical visits, telephone calls, and indirect contacts such as letters, with physicians as well as nurses. The data provided from each PHCC was extracted as the total number of ICD-10 diagnosis codes per month, and no individual level data was used.

Descriptive data about the PHCCs was collected from the regional central health care register, including number of patients listed at each PHCC, gender and age distribution of patients, number of staff members, and the CNI and the ACG for the patient population of each PHCC.

### 2.4 Study variables

We used the outcome variables *alcohol-related ICD-10 codes* as a proxy measure of the number of alcohol-related physician consultations. The alcohol-related ICD-10 codes extracted from the medical records were F10.0-F10.9 (mental and behavioral disorders due to use of alcohol).

The predictor variables we used were the following: *PHCC* (intervention or control), *month* (we used monthly periods and not calendar months, since the intervention started on different dates at the two PHCCs), *PHCC x month* (the interaction term between PHCC and month), *intervention (level)*, *intervention (slope)*, *education (level)* and *education (slope)*. Since we could not know in advance if the effect of the intervention would be instantaneous or gradual, we constructed two variables to describe the intervention effect. *Intervention (level)* models a “level change”, i.e. an instantaneous increase or decrease in the number of ICD-10 diagnosis codes shortly after the start of the intervention, with a subsequent plateau at a new level. *Intervention (slope)* models a “slope change”, i.e. a gradual increase or decrease in the number of ICD-10 diagnosis codes during the intervention period. We also constructed two variables to control for a possible effect of the educational lecture that both PHCCs received. These were constructed in the same way as the intervention variables, i.e. one for level change and one for slope change.

### 2.5 Statistical analyses

The analyses were conducted in R version 3.3.2 (R Core Team (2015), 2015). We analysed the data using an interrupted time series (ITS) design with a control group. An interrupted time series analysis is a quasi-experimental analysis in which the effect of an intervention or event is assessed by using time-series data before and after the intervention or event. The basic idea of ITS is that the time series data before the intervention has a particular level and slope (corresponding to mean and trend, respectively, within the linear equation framework), and the intervention can affect either the level or the slope, or both. A statistically significant effect of the post-intervention level or slope change indicates an effect of the intervention. Data from a control group can be used in order to strengthen the analysis. In this case, each group may have its own base level and slope, and the intervention will just affect the intervention group. In the present study, there was an added complication in that both PHCCs received an educational lecture, which within the ITS framework can be considered as an additional intervention. We therefore treated the eductional lecture as a separate intervention and thus used an ITS analysis with two interventions (the “nurse intervention” and the “educational lecture intervention”) and a control group, in which the “educational lecture intervention” was given at both PHCCs and the “nurse intervention” (i.e. the actual intervention of the study) was given at the IPHCC but not at the CPHCC.

The ITS models were analysed using Poisson regression because the outcome variable was defined as the number of ICD-10 diagnosis codes. We used the total number of patient consultations each monthly period as an offset variable, so that the outcome variables reflect the rate of diagnosis codes per 1000 patient contacts.

A series of eight different statistical models were computed, including all possible combinations of *education (level)* or *education (slope)*, *intervention (level)* or *intervention (slope)*, and the inclusion or not of *PHCC x month*. The best-fitting model was selected using the Akaike information criterion (AIC). This final model, which included the predictor variables PHCC, month, PHCC x month, education (level), and intervention (slope), had an AIC value of 230.0 For the main analyses we conducted quasi-Poisson regression analyses, to take into account the overdispersion that can be seen in a Poisson distribution. The estimated dispersion parameters for the final models are presented. We could not find any signs of significant autocorrelation. Analysis of the residual charts did not show any visual signs of heteroscedasticity. Rate ratios (RR) of the outcome events are reported with 95 % confidence intervals (CI).

## 3 Results

At the IPHCC, the mean number of registered alcohol-related ICD-10 codes per month was 1.7 during the year before study start, and 3.0 during the year in which the study was conducted, corresponding to an increase of 76 %. At the CPHCC, the mean number of registered alcohol-related ICD-10 codes per month increased from 6.0 before the study start to 15.1 during the study period, corresponding to an increase of 152 %.

**Fig. 1:**
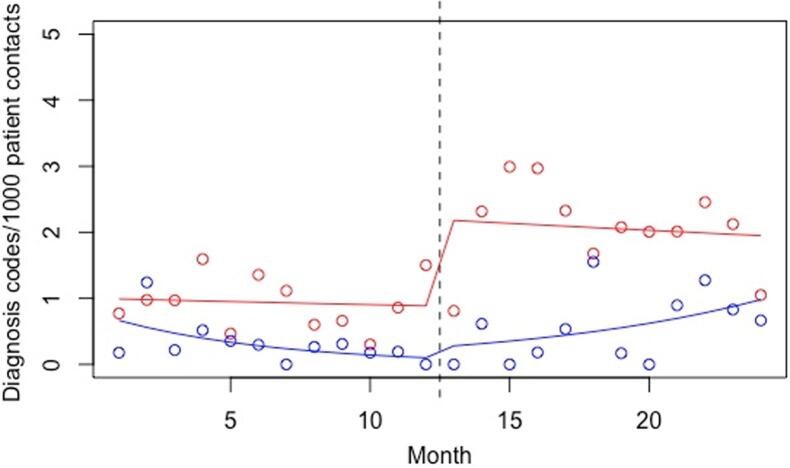
Diagram over the number of registered alcohol-related ICD-10 codes (main analysis). The colored lines represent the predicted values from the Poisson regression models. The blue line illustrates the intervention PHCC and the red line illustrates the control PHCC. The dotted line in the middle illustrates the start of the intervention. (For interpretation of the references to colour in this figure legend, the reader is referred to the web version of this article.)

In the main statistical model, selected for having the best model fit by the AIC, the effect of the intervention was modelled as a slope effect and the effect of the educational lecture was modelled as a level effect, and the PHCC x month variable modelled the differential baseline trajectories of the two PHCC’s (Table 2). As can be seen in Fig. 1, the baseline level of alcohol-related ICD-10 codes was not significantly different at the IPHCC (RR: 0.74, 95 % CI 0.32–1.68). The general trend over time was non-significant at 0.99 per month, but the interaction between PHCC and month (PHCC x month) was significant at a RR of 0.86 (95 % CI 0.78–0.95), meaning that there was a baseline declining trend in the IPHCC that was not present in the CPHCC. The different baseline level and trend between the two PHCC’s can be seen in the left part of Fig. 1, before the line indicating the time point of the intervention.

**Table 2:** Factors associated with an increase in the number of registered alcohol-related ICD-10 codes (main analysis).

Variables	RR	95 % CI	p-value
IPHCC	0.74	0.28–1.94	0.54
Month	0.99	0.95–1.03	0.62
PHCC x month	0.86	0.76–0.97	0.02
Education (level)	2.47	1.37–4.46	<0.01
Intervention (slope)	1.33	1.08–1.62	0.01

IPHCC represents the difference in mean value for the PHCCs, month represents the general trend at the PHCCs, PHCC x month represents the difference in trend between the PHCCs, education (level) represents the instantaneous effect of the educational lecture and intervention (slope) represents the gradual effect of the intervention.

RR = rate ratio, CI = confidence interval.

The educational lecture, modelled in the main statistical model as a level change, was significantly associated with an increase in the number of registered alcohol-related ICD-10 codes (RR: 2.47, 95 % CI: 1.37–4.46), which is clearly visible in Fig. 1. Finally, the intervention was significantly associated with an increase in the number of registered alcohol-related ICD-10 codes per month (RR: 1.33, 95 % CI 1.08–1.62), which is shown in Fig. 1 as the upward slope for the IPHCC following the intervention time point.

In the remaining seven statistical models, the intervention was not significantly associated with the outcome in any of the four models in which it was modelled as a level change. In one of the remaining three models, in which the intervention was modelled as a slope change, it was significantly associated with an increase in the number of registered alcohol-related ICD-10 codes.

The educational lecture was significantly associated with an increase in the number of registered alcohol-related ICD-10 codes in all of the three models in which it was modelled as level change. In the remaining four models, in which the educational lecture effect was modelled as a slope change, it was not significantly associated with the outcome.

## 4 Discussion

The main aim of this study was to investigate whether the presence of a specialized nurse with experience from addiction healthcare would increase the number of alcohol-related physician consultations in a PHC setting. The results from our main analysis suggest an increase in the number of registered alcohol-related ICD-10 codes in both the IPHCC and the CPHHC after the intervention, with a clear and momentary association at both units between the educational lecture and increased alcohol-related codes, as well as a more association by the intervention proper in the IPHCC. However, when taking the results of the sensitivity analyses into account, the association with the intervention seems less robust, such that the association between the intervention and a subsequent increase in the number of registered alcohol-related ICD-10 codes seems uncertain. Thus, while the association between the lecture and a favorable outcome is a secondary finding seen at both units, the gradual association with the intervention studied (the presence of a nurse specialized in AUDs at a primary care unit) was promising but less certain. While the present findings hold promise for specialized interventions within primary care, more research is needed in order to further test its actual effects.

Interestingly, the marked association between the educational lecture given at both PHCCs and an increase in the number of registered alcohol-related ICD-10 codes was statistically significant, and this finding remained in all the analyses in which the education was modelled as a momentary effect (level change), but not in any of the models where it was modelled as a more gradual effect (slope change). The educational lecture was thus associated with an instantaneous increase in the number of registered alcohol-related ICD-10 codes. Our interpretation of this finding is that the educational lecture might have led to increased awareness of and attention to AUDs, resulting in increased screening and treatment of these disorders. This association was significant at both units. However, the reasons behind the fact that it was even larger, in absolute numbers, at the control unit, cannot be investigated in the present study, but may be related to baseline levels of alcohol screening or baseline levels of knowledge and routine work in the staff.

The effectiveness of different methods for implementing alcohol interventions in PHC seems to correlate with the intensity of the intervention effort, i.e. the amount of training and/or support provided (Nilsen, Aalto, Bendtsen, & Seppä, 2006). Previous studies have found that more training and support for healthcare providers in PHC increases the screening and intervention activity (Anderson, Kaner, & Keurhorst, 2017). In the present study, the main intervention was the recruitment of a nurse from an addiction clinic who was present at the IPHCC 20 h per week. Additionally, the staff at both PHCCs received a single educational lecture about the screening, diagnosing and treatment of AUDs. While we did not find a clear association between the presence of the study nurse and an increase in alcohol-related physician consultations, the results of the study did imply that providing a single educational lecture to PHC staff in the screening and interventions for AUDs could be a more effective way to increase the diagnosis and treatment of these disorders.

Several limitations of this study need to be considered. First, this was a small study solely including one IPHCC and one CPHCC. Both PHCCs were located in the inner-city area of the same city, but the CPHCC had a larger number of patients and staff. Furthermore, the IPHCC had a larger proportion of male patients compared to the CPHCC, as well as a higher ACG (implying a higher degree of total morbidity among patients). These differences might have affected the results. Considering the outcome variable, the number of registered alcohol-related ICD-10 codes is only an indirect measurement of the outcome we intended to study, i.e. an increase in alcohol-related physician consultations. For some of the physician consultations with a registered alcohol-related ICD-10 code, this might only have been a secondary diagnosis code, and the consultation might not have been related to an AUD. As the study measured the level of activity in diagnosing alcohol use disorders overall, as a potential effect of the interventions, it cannot be fully outlined whether diagnostic codes represent an increased uptake of new patients or an increased tendency to diagnose patients already in treatment at the unit. Furthermore, we were only able to obtain data on the ICD-10 codes registered by physicians at the two PHCCs, wherefore ICD-10 codes registered by other staff members were not included in the analyses. In addition, although staff were not followed in outcome data on an individual level, and it is unlikely that staff would have experience a risk of being surveilled individually, it still cannot be excluded that awareness of the study itself increased the diagnostic activity in the staff of both units.

By including the outcome variables in the analyses as fractions of the total number of patient contacts each monthly period, we were able to control for possible effects of changes in total activity at the PHCCs. By including the month variable as an independent variable, we were further able to control the analyses for underlying time-dependent changes in the registration of ICD-10 codes that were independent of the intervention. We were however not able to control for other variables that might have affected the results.

Taking these limitations into consideration, our findings should be interpreted with caution. Keeping this in mind, the present study did show an increase in registered alcohol-related ICD-10 codes at both the intervention and the CPHCC. Our findings thus suggest that an educational lecture on alcohol problems and AUDs, which was given at both PHCCs, can increase the number of alcohol-related physician consultations in a primary care setting. These findings indicate the value of knowledge about AUDs to increase the activity of alcohol interventions. The educational lecture might thus have led to a general awareness about alcohol problems and AUDs amongst the staff at both PHCCs, and perhaps motivated them to be more active in the screening for these conditions. The increase in the number of registered alcohol-related ICD-10 codes was however larger at the CPHCC, and the association between the main intervention, i.e. the presence of a specialized alcohol nurse, and an increase in registered alcohol-related ICD-10 codes was uncertain. Thus, the mere presence of the study nurse does not seem to have increased the detection of alcohol-related diagnoses among the physicians at the IPHCC, above and beyond the association with the educational lecture. These results might partly be explained by the limitation that the study nurse’s registered diagnosis codes were not included in the analyses. It is possible that the study nurse took care of many patients with alcohol problems and AUDs that would have otherwise been referred to the physicians at the IPHCC.

### 4.1 Conclusions

The fact that a majority of individuals with AUDs do not receive medical care (Socialdepartementet, 2011) needs to be better addressed by the health care system. PHC has been suggested as an important arena for screening and initial treatment (Socialdepartementet, Government of Sweden, 2011, Carvalho et al., 2019, Rehm et al., 2016). The present study proposed a method to increase the number of alcohol-related physician consultations in PHC, by providing a PHCC with a specialized addiction resource. The findings of the present study were however uncertain, and larger studies are needed to conclude whether the presence of specialized addiction staff in PHC can increase the number of alcohol-related physician consultations. The results of the present study do however imply that a single educational lecture about harmful alcohol use could be a simple but effective intervention to increase the screening for and treatment of these disorders for several months following such an intervention.

## Funding

This study was funded by the Swedish Alcohol Monopoly Research Council (Systembolagets Alkoholforskningsråd, SRA) and the 10.13039/501100007687Swedish Society of Medicine (Svenska Läkaresällskapet), the Söderström-Königska foundation.

## CRediT authorship contribution statement

**Tove Abrahamsson:** Data curation, Formal analysis, Investigation, Methodology, Validation, Visualization, Writing – original draft. **Ester Magnusdottir:** Data curation, Formal analysis, Investigation, Methodology, Validation, Visualization, Writing – original draft. **Jonas Berge:** Data curation, Investigation, Methodology, Software, Supervision, Validation, Writing – review & editing. **Åsa Lundvall:** Conceptualization, Data curation, Investigation, Project administration, Writing – review & editing. **Agneta Öjehagen:** Conceptualization, Funding acquisition, Investigation, Methodology, Project administration, Resources, Supervision, Validation, Writing – review & editing. **Anders Håkansson:** Conceptualization, Data curation, Funding acquisition, Investigation, Methodology, Project administration, Resources, Supervision, Validation, Writing – review & editing.

## Declaration of competing interest

The authors declare that they have no known competing financial interests or personal relationships that could have appeared to influence the work reported in this paper.

## Data Availability

The authors do not have permission to share data.
